# Salts of Therapeutic Agents: Chemical, Physicochemical, and Biological Considerations

**DOI:** 10.3390/molecules23071719

**Published:** 2018-07-14

**Authors:** Deepak Gupta, Deepak Bhatia, Vivek Dave, Vijaykumar Sutariya, Sheeba Varghese Gupta

**Affiliations:** 1 Department of Pharmaceutical Sciences, School of Pharmacy, Lake Erie College of Osteopathic Medicine, Bradenton, FL 34211, USA; dgupta@lecom.edu; 2 ICPH Fairfax Bernard J. Dunn School of Pharmacy, Shenandoah University, Fairfax, VA 22031, USA; dbhatia@su.edu; 3 Wegmans School of Pharmacy, St. John Fisher College, Rochester, NY 14618, USA; vdave@sjfc.edu; 4 Department of Pharmaceutical Sciences, USF College of Pharmacy, Tampa, FL 33612, USA; vsutariya@health.usf.edu

**Keywords:** chemistry, salt, water solubility, routes of administration, physicochemical, stability, degradation

## Abstract

The physicochemical and biological properties of active pharmaceutical ingredients (APIs) are greatly affected by their salt forms. The choice of a particular salt formulation is based on numerous factors such as API chemistry, intended dosage form, pharmacokinetics, and pharmacodynamics. The appropriate salt can improve the overall therapeutic and pharmaceutical effects of an API. However, the incorrect salt form can have the opposite effect, and can be quite detrimental for overall drug development. This review summarizes several criteria for choosing the appropriate salt forms, along with the effects of salt forms on the pharmaceutical properties of APIs. In addition to a comprehensive review of the selection criteria, this review also gives a brief historic perspective of the salt selection processes.

## 1. Introduction

The specific salts of active pharmaceutical ingredients (APIs) are often formed to achieve desirable formulation properties. Although addressing poor aqueous solubility is one of the most important reasons to employ a salt formation, pharmaceutical companies also use the formation of unique salt products to commonly address other physicochemical and biological concerns such as stability, toxicity, poor absorption, and issues related to manufacturing processes. The importance of salts is indicated by approximately 50% of the United States Food and Drug Administration (US FDA) approvals consisting of APIs in the salt form [1]. Moreover, half of the top 200 prescription drugs in the United States consist of pharmaceutical salts [2]. The choice of the appropriate salt form is dictated by various factors. The formation of potentially marketable salt requires concerted efforts and a thorough understanding of the physical and chemical characteristics of the API and counterions that are used. A rational decision tree approach should be followed for the selection of the best salt in the most economical way. Furthermore, all of the necessary testing should be performed in the early phases of the drug development process in order to minimize failures. Salts can significantly alter physical/chemical properties of an API so much so that it can expedite the drug development process.

The suitability of a candidate for salt selection is determined by the physical and chemical properties of the API; different counterions can be utilized to address one or more shortcomings of the API. The prediction of a salt’s qualitative and/or quantitative properties based on the counterion used is an important research area. Several studies have described a link between salt properties and the counterions used [3,4,5,6,7,8]. While predictions can be made with some degree of accuracy, there is no reliable way to accurately investigate salt properties based on the counterion used. Currently, a wide range of validated counterions is available to prepare the salts of APIs (Table 1) [9]. One important criterion in the selection of counterions is to employ agents that have been previously used in FDA-approved drugs, and are thereby generally recognized as safe (GRAS) [7].

This review will address various criteria for the selection of salt forms, as well as suitable examples for each category. Inclusion of all of the examples for each criterion will be beyond the scope of this review; therefore, only a few representative examples are included. It should be noted that various textbooks have been published addressing the salt forms of API; the focus of the majority of the literature is the enhancement of API solubility through salt formation. This review is unique, and aims at offering a succinct report on the salt selection criteria based on the chemical, pharmaceutical, biological, and economical applications of different salt formulations.

## 2. Drug Chemistry Considerations

### 2.1. API Functional Groups

The presence of acidic or basic functional groups is an essential requirement for the formation of salts. A majority of the APIs discovered are suitable candidates for salt formation during drug development, since they are either weakly acidic or weakly basic in nature. Salt screening begins with the characterization of acidic or basic functional groups. Depending on the presence of these groups and pharmaceutical needs, a potential counterion can be selected. Low molecular weight bases and acids have higher chances of being a liquid with a low melting point. Salt formation can be employed to augment their melting points and convert and maintain the solid state. For example, Bozigian et al. reported that compound NBI-75043, which is an investigational compound for the treatment of insomnia, was a crystalline, free base with a low melting point (64 °C) [10]. One of the important pharmaceutical requirements for this compound was to develop a salt that possessed a higher melting point. Since weakly basic drugs require acidic counterions to form ionic bonds, 14 acids were selected as possible counterions. Since the low melting point was one of the concerns for this drug, initial approaches to characterize salt forms included differential scanning calorimetry (DSC), which is an important tool for determining the melting point as well as crystallinity, solvates, and presence or absence of the polymorphs. They were able to successfully find the salt form of NBI-75043 by focusing on the chemistry of the drug [10].

### 2.2. pK_a_ of the Drug

The selection of a counterion is based on the pK_a_ rule, which takes into account the degree of ionization of the acidic or basic functional groups that are present in the drug [11]. According to the pK_a_ rule, when the pK_a_ difference between an acid and base is greater than two or three, salt formation is expected [11,12]. Ideally, for basic drugs, the pK_a_ should be at least two pH units higher than the pK_a_ of the counterion, and for acidic drugs, the pK_a_ of the drug should be at least two pH units lower than the pK_a_ of the counterion chosen. This difference ensures strong binding energy between the opposite ionic species so that the complexes formed will not readily break down into individual species when not required. For example, phenytoin is a well-known acidic drug with a pK_a_ value of 8.4; however, it has limited solubility. One important pharmaceutical property for this drug that needed to be addressed was improving its aqueous solubility. Due to the acidic nature of the drug, basic counterions with pK_a_ values >10.4 were likely to form pharmaceutically acceptable salts. Therefore, a strong basic counterion such as NaOH was needed to form a desirable salt of phenytoin. Weakly basic counterions would not be able to form salts with phenytoin, since these counterions would not be able to raise the pH above the required pH_max_ value of 11 [13].

### 2.3. Lipophilicity

Salt formation is a well-utilized technique to increase the aqueous solubility of a drug. However, hydrophobic salt approaches are sometimes considered to increase the lipophilicity of a drug molecule [14,15]. The decrease in aqueous solubility has been found to be a useful approach to provide greater chemical stability, particularly at high humidity and high temperature. One well-known example is the formation of sulfate as well as hydrophobic salts of xilobam. The sulfate salt of this drug is completely ionized. In fact, it has been found that the presence of aryl groups in the sulfate counterion for this drug protected the base from getting easily hydrolyzed in the presence of high humidity and high temperatures. The formation of hydrophobic salts allows pharmaceutical companies to prepare more stable drugs without affecting their bioavailability [16]. Salt formation leads to increased lipophilicity as a result of the neutralization of the overall electrostatic charge, thereby enhancing the membrane permeability of hydrophilic molecules. As shown in Table 2, Sarveiya et al. correlated the effect of several counterions of ibuprofen on log P value and membrane absorption [17], and clearly demonstrated the effects of the different counterions on these properties.

### 2.4. Hygroscopicity

Hygroscopicity is defined as the ability of a material to absorb and retain moisture at various temperatures and humidity conditions. Low hygroscopicity is a preferred characteristic of drugs, as the moisture content can significantly affect stability. Based on the extent of water uptake, APIs can be classified as non-hygroscopic, slightly hygroscopic, and hygroscopic solids [18]. A non-hygroscopic substance can take up moisture from a humid environment, which in turn can alter the mechanical and solubility properties, affecting the performance of a drug. Readily hydrolyzable drugs are more easily degraded due to the presence of water and pH alterations in the microenvironment of the salt. Thus, hygroscopicity needs to be carefully monitored when designing a salt form of a drug. For example, the salts of mineral acids tend to be very polar, leading to increased hygroscopicity and low microenvironmental pH. These factors can affect the stability of some drugs due to a consequential increase in the rate of hydrolysis [19].

### 2.5. Water of Hydration

A salt with the associated water of crystallization is considered as a hydrate form. These forms have water molecule(s) in the crystalline lattice of the API. Hydrate forms of APIs are quite common; it is estimated that approximately one-third of APIs can form hydrates if exposed to the conditions that are conducive for hydrate formation [20]. Pharmaceutical hydrates are formed when the API comes in contact with water during crystallization, lyophilization, wet granulation, aqueous film coating, spray drying, and storage [21]. If a hydrate is exposed to a dry environment, it can lose the water of crystallization to attain a lower state of hydration or an anhydrous form. The exchange of water between drug and excipients such as starch or cellulose can also affect the solubility and mechanical properties of a drug product [22,23]. Water molecules in pharmaceutical hydrates influence the internal energy, thermodynamic activity, hygroscopicity, solubility, dissolution rate, and stability [23]. Therefore, understanding the hydrate form is crucial in order to better understand these properties and address significant issues if the need arises.

### 2.6. Polymorphism

Polymorphism is the ability of a solid compound to exist in more than one crystalline form. Most drugs exhibit structural polymorphism or multiple crystalline forms. In order for a molecule to develop into a potential drug, the existence of a stable polymorph or a suitable pseudopolymorph needs to be established. The polymorphs (or pseudopolymorphs) of drugs show different chemical stability; it is generally observed that a more thermodynamically stable polymorph is more chemically stable than a metastable polymorph [24]. The optimized orientation of molecules, hydrogen bonds, and non-hydrogen bonds in the crystal lattice play an important role in imparting thermodynamic stability to crystal structures. Even small changes in the crystal packing may lead to significant differences in the chemical reactivity of the two polymorphs of the same drug [24]. Between the crystalline form and amorphous forms of the same drug, the amorphous form is less stable due to the lack of a three dimensional crystal structure, free volume, and greater molecular mobility [24]. The amorphous form of penicillin G was shown to be less stable than the crystalline sodium and potassium salts [25]. There are several examples of drug polymorphism’s effects on the pharmaceutical fate of the drug. It is beyond the intended scope of this review to address all of the examples. However, it is worthwhile to mention the polymorphism of ritonavir (Norvir^®^), the discovery of which served as a wake-up call for the pharmaceutical companies. Ritonavir is an antiviral drug marketed by Abbott Laboratories in 1996 in the form of semisolid gel capsules for the treatment of acquired immunodeficiency syndrome (AIDS) [26]. The capsules contained the only known crystal form, Form I, which was discovered during the development process. However, in 1998, a new and significantly less soluble polymorph of ritonavir precipitated in the semisolid gel capsules [27,28], which became known as Form II. This form demonstrated a significantly lower solubility in hydroalcoholic solutions than the marketed Form I [28]. The manufacturing of ritonavir semisolid capsules formulation was comprised of a hydroalcoholic solution of the drug, which was found to be saturated with Form II. The sudden appearance and dominance of this less soluble form made the formulation unmanufacturable [27], and also affected the storage of Norvir^®^ oral solution at refrigeration conditions, since lower storage temperatures led to the crystallization of Form II [27]. These factors, along with limited inventory, led to the withdrawal of the drug by Abbot Laboratories, leaving tens of thousands of AIDS patients around the world without medication [26]. Ritonavir was reformulated and approved in 1999 before being placed on the market; Abbot lost revenue of over US $250 million in the process [26]. Therefore, understanding salt formulations and their correlation to polymorphism early in drug development is imperative to minimize drug failures at later stages of drug development.

### 2.7. Chemical Stability

Acidic or basic counterions can alter the pH of the microenvironment in liquid dosage forms. In turn, changes in pH can influence the reactivity of an API with excipients, and can lead to either the improved stability or degradation of the API. Undesirable interactions can generate significant impurities in a drug product [29].

**Figure 1:**
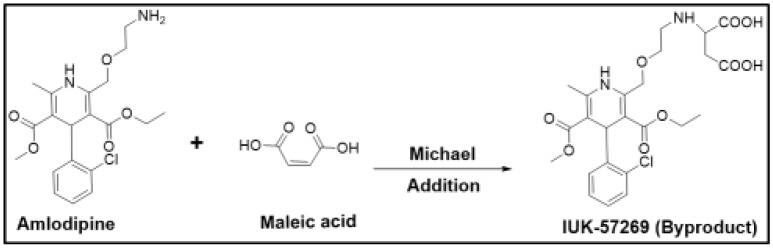
Degradation reaction of amlodipine in the presence of maleic acid.

For example, amlodipine is a free base that was initially chosen for developing a maleate salt. However, the presence of maleic acid changed the microenvironment of the drug product, and this alteration led to the formation of the aspartic acid derivative (UK-57269) by Michael addition, as shown in Figure 1. This degradation product was found to have different biological activity, and therefore, amlodipine maleate was found to be unsuitable for further development. Although such reactions could be minimized by the careful selection of excipients and by avoiding alkaline conditions [30], besylate (benzenesulfonate) was chosen to be the suitable salt form with significantly fewer problems [12]. This example clearly demonstrates how drug stability can be adversely affected if a counterion is not carefully chosen.

### 2.8. Solubility and Dissolution Rate

Salt formation approaches have widely been utilized to increase solubility, and therefore, the dissolution rate of a drug. It is one of the most common methods to increase the solubility of weakly acidic and basic drugs. Hydrochloride, mesylate, hydrobromide, acetate, and fumarate are the most common counterions that are used for basic chemical entities in the past 20 years [31], while sodium, calcium, and potassium continue to be the most common counterions for weakly acidic drugs. Increases in aqueous solubility have been achieved by most of these counterions. Slater et al. studied the feasibility of salt formation for RPR2000765, having a pKa of 5.3 and an intrinsic free base solubility of 10 µg/mL [32]. The poor aqueous solubility yielded poor bioavailability in animals. While all of the salt forms (hydrochloride, hydrobromide, methanesulfonate, mesylate, and camphorsulfonate) increased the solubility of the parent drug, mesylate salt consistently produced a higher solubility of 39 mg/mL at 25 °C. Other factors such as hygroscopicity, clean polymorphic profile, particle size, and flow properties were also considered, and all of these factors favored the formation of a mesylate salt for further development [32]. This shows that the selection of a suitable counterion should not be an isolated approach that focuses on one consideration at a time, but should instead be a holistic approach, incorporating additional relevant considerations simultaneously.

## 3. Pharmaceutical Considerations

### 3.1. Dosage Form Desired

#### 3.1.1. A Liquids (Suspensions)

Suspensions are the most common type of oral liquid dosage forms. Masking the taste of bitter drugs is one of the important considerations during drug formulation development. The use of different counterions to make suitable salt forms has been utilized effectively to either make suspensions or reduce solubility so that drugs do not dissolve well when placed on the tongue. For example, erythromycin (free base) is a well-known macrolide antibiotic that is freely soluble in water. However, higher solubility leads to faster dissolution on the tongue, leading to a bitter taste. This unique characteristic was found to be a great deterrent for pediatric formulations. Salt formulations were later sought to reduce solubility. Of the various salt forms screened, stearic acid salt was found to have reduced solubility, and further allowed the formulation of a suspension that effectively suppressed the bitter taste of the free base. This makes the acidic salt form of erythromycin much more pharmaceutically acceptable, especially in pediatric patients. Similar to erythromycin, in order to reduce the solubility of an acidic or basic drug, salts can be synthesized to allow the development of a suspension formulation. For acidic drugs, calcium salts or anion exchange resonates can be considered. For basic drugs, the salts of long chain fatty acids (e.g., laurates and pamoates) and cation exchange resonates can be a good choice [12].

Similarly, sweeteners such as cyclamic acid or saccharin can be useful to make salts for basic drugs. In case of acidic drugs, basic salts such as triethanolamines can be useful for improving the taste [12]. These examples demonstrate that salt strategies may be an effective approach to mask the taste of bitter drugs.

#### 3.1.2. B Solutions

The solubility of a drug in aqueous systems is an important factor in the development of parenteral formulations. Thus, in order to increase solubility, salt forms are frequently employed to make concentrated parenteral solutions. Appropriate counterions can be straightforwardly screened based on solubility experiments, which serve as an important tool for finding the best candidate. In most cases, solubility can be increased by altering the pH of a solution. One well-known example is phenytoin sodium, in which the solubility is significantly increased by the addition of sodium hydroxide (NaOH), in order to allow parenteral administration at a desired concentration [33]. Chemical stability is another crucial factor, as drugs in solution tend to be less stable than in solid dosage forms. For example, cephalosporin antibiotics are neutral zwitterions, and are not very stable in solution. Mono-counterion salts did not offer much stability, and although di-counterion salts yielded stable solutions, they were quite acidic with pH < 2. This pH problem was resolved by preparing a di-hydrochloride salt to be reconstituted with 2 mL of arginine at the time of injection. This led to a stable drug solution within a desired pH range [12]. This clearly reflects that salt formulations can be exploited to make appropriate parenteral solutions for a desired therapeutic outcome.

#### 3.1.3. Creams/Ointments/Gels

Creams, ointments, and gels are commonly used dosage forms for transdermal delivery. Highly polar transdermal drug candidates generally demonstrate ineffective percutaneous penetration [34]. This limits the use of some important drugs. Salt formation has been utilized in the past to increase transdermal permeability. Counterions act as neutralizing agents by binding with the API via Coulomb forces to permit passive absorption. For example, the ion-pairing of salicylates with alkylamines and quaternary ammonium ions showed an increase in the percutaneous flux of the drug. Increased penetration was successfully attained with the diethylamine salt of diclofenac as a topical gel, while the sodium salt is available for oral absorption [35]. Therefore, a suitable formulation can be developed to increase transdermal permeability for desired systemic effects.

#### 3.1.4. Aerosols

The inhalation route is primarily targeted to bronchioles and lungs for local drug delivery, but various physiochemical and mechanical factors should be considered for effective delivery. The limited residence time of the drug at the site of action is one of the most common and important barriers to the effective utilization of APIs. Salt formulations have been instrumental in providing the local delivery of drugs with much longer half-lives. For example, salmeterol is a long-acting beta adrenergic agonist that is used in the therapy for chronic obstructive pulmonary disease (COPD). Reduced solubility was necessary to allow for a longer time at the site of action, and was accomplished by the development of an xinafoate salt. This slow-dissolving compound potentiated the long half-life of salmeterol. Therefore, the xinafoate salt of salmeterol served as an important formulation and a prime example to demonstrate how the properties of APIs can be modified for desired outcomes [36].

### 3.2. Ease of Synthesis and Scale-Up

#### 3.2.1. Flowability

Generally, an API with decent flow properties is considered a good candidate for the development of commercially successful solid oral dosage forms. The flowability of the drug can have significant effects on the blending, compression, filling, transportation, and scale-up operations of solid dosage manufacturing. APIs with poor flow properties may result in final products with unacceptable uniformity content, weight variation, and physical inconsistency. The crystalline nature of an API is mostly preferred, as it is amenable to techniques that improve flow properties [37]. Thus, an amorphous drug can be formulated into a suitable salt form that improves its solid state properties by promoting a crystalline structure.

#### 3.2.2. Corrosiveness of Counterions

A drug with a highly corrosive nature can be a significant barrier to a successful manufacturing process. Although utilizing a counterion approach to mask the corrosive group can solve the problem to a greater extent, if selection is not done well, it can lead to more problems in the latter parts of drug development. For example, weakly basic drugs with low dissociation constant (pKa) values require salts of much stronger counterion acids to be physically stable [38]. This may lead to acidic aqueous solutions of the salt. Highly acidic aqueous solutions can corrode metal containers, manufacturing tools, and other equipment. Therefore, parts used for tableting such as punches, dies, and die tables are more vulnerable to the damage caused by corrosive solids, since they are in continuous contact with tablet mixtures under high pressure and friction. The metal surface of capsule-filling machines and mechanical forces involved in filling medications can also become corroded. Corrosive salts can make tableting technically impossible, and if used, may lead to metal traces in tablets during compression. Consequently, these types of corrosive counterions should not be used to create salt formulations, or alternatively, should be sufficiently diluted with excipients to avoid serious corrosive properties [39]. Furthermore, the salts of drug products with pH values of 2.5 or lower for saturated aqueous solutions are generally found to be corrosive. Corrosiveness tests should be conducted if the pH value of a saturated aqueous solution is less than or equal to four. For example, weakly basic drugs (pKa = 4.7), as mentioned by Stahl et al., were considered to be developed as either free bases or hydrochloride/methanesulfonate salts. However, the hydrochloride salt was later dropped due to extreme corrosiveness. The methanesulfonate was not corrosive on stainless steel, and was only slightly corrosive on grey cast iron and tool steel alloys. Therefore, methanesulfonate was chosen as the preferred counterion, followed by further development [12]. Thus, the unique properties of counterions need to be studied in order to properly to manufacture non-corrosive drug products.

#### 3.2.3. Compatibility with Excipients

There are numerous examples in the literature regarding the selection of suitable salt forms to minimize the interaction of APIs with various excipients, thereby making the drug product chemically feasible to develop. The selection of the counterion should be based on an understanding of the types of chemical interactions with the excipients.

**Figure 2:**
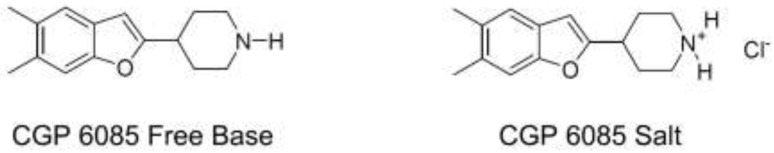
Free base and salt form of CGP6085.

For example, the free base form of compound CGP6085 was initially designed as an antidepressant. However, its unwanted interactions with the tablet excipient, lactose led to the significant degradation of the API (Figure 2). Thus, the free base form was found to be a non-viable option. The hydrochloride salt form of CGP6085 was later developed, which significantly improved the stability of the API, and eventually suppressed interaction with lactose [12]. This example illustrates how salt forms can have a significant influence on drug stability, and unique counterions have the potential to increase the stability of a drug in the chosen dosage form.

### 3.3. Route of Administration

Routes of drug administration dictate the use of the free form (acid/base) of a drug, or whether a suitable salt form is warranted. In fact, for some drug formulation types, it is even more important to have a salt form than a non-salt form. One of the major concerns for injectable dosage forms is the limited solubility of a drug in the few vehicles that are suitable for injection. For example, the formation of salt formulations is much more crucial for injectable dosage forms than oral or transdermal dosage forms. This is because injectable drugs, which are mainly intravenous (IV), require soluble products to avoid phlebitis or tissue irritation due to insoluble therapeutic agents [40]. Historically, more injectable salt forms have been approved than any other salt forms. A review article by Paulekuhn et al. described how more than 70% injectable dosage forms that contained salts as compared with only 50–60% of oral dosage forms. A greater need for a highly soluble salt for injectable dosage forms is one of the important driving forces behind salt forms [1]. The most commonly used anions for oral dosage forms are chloride, sulfate, and maleate; chloride, sulfate, and acetate were the three top anions used for injectable dosage forms. Whereas, sodium, potassium, and calcium were the three most favored cations used for both oral and parenteral formulations. Recently, the lysine counterion has become a popular choice for injectables, appearing in approximately 15% of injectable salts that were approved between 2002–2006 [1]. Thus, salt formation is one of the important ways to achieve the desired characteristics in a drug, such as increased solubility for the parenteral route of administration.

Different salt forms of the same drug can be suitable for different routes of administration as well. For example, sodium, potassium, and the free acid forms of diclofenac have been approved as oral medications. Diclofenac sodium 1% gel (Voltaren Gel^®^) and diclofenac sodium topical solution 1.5% *w*/*w* (Pennsaid^®^) are also available as topical products; however, its epolamine salt (Flector^®^) is approved as a transdermal patch due to its better skin permeation than sodium or potassium salts [41].

### 3.4. Controlled Release Dosage Forms

**Figure 3:**
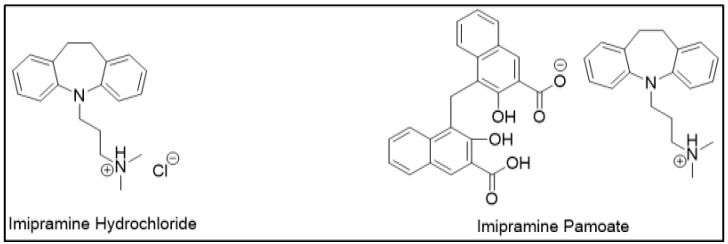
Salts of imipramine.

APIs demonstrate different dissolution and release properties when attached to different counterions; this property has been utilized to design controlled-release drug forms. Clinically, one salt form may be preferred over another for desired release characteristics. For example, a highly soluble drug can be designed into a controlled release formulation by using sparingly soluble salts. This decrease in drug solubility may retard the drug release as desired. Therefore, selecting an appropriate counterion to slow down drug release can be helpful in sustained release (SR) formulations. For example, imipramine, which is a tricyclic antidepressant, was initially designed as hydrochloride salt as an immediate release (IR) formulation (Figure 3). However, a controlled release formulation was more desirable to maintain sustained therapeutic effects. For the same reason, imipramine pamoate was designed, and its solubility was found to be significantly less than hydrochloride salt. This delay in the rate of drug release was suitable for the desired SR formulation [12].

Another example that illustrates the importance of dissolution is the various salt forms of diclofenac. Fini et al. examined the dissolution of 30 different salt forms of diclofenac [42]. While both potassium and free acid form are now being used for the immediate release dosage form in the US, only the sodium salt form is used for the extended release dosage form. Thus, different counterions that are attached to the same drug can influence dissolution rates, and therefore can influence dosage forms desired in clinical practice.

## 4. Pharmacokinetics (PK), Pharmacodynamics (PD), and Safety Considerations

### 4.1. Toxicological Consideration

When a drug is administered orally, it has the potential to interact with the gastrointestinal (GI) wall lining, causing unwanted side effects. This type of toxicity concern with APIs is crucial, and can be instrumental in limiting the effective utilization of drugs. At times, salt approaches have been utilized to reduce the gastrointestinal (GI) toxicity of the parent drug. Various examples [43,44,45] demonstrate this use of counterions that were readily metabolized and excreted while having minimal toxicity concerns. Thus, these approaches were helpful in addressing GI toxicity concerns. For example, salicylates are known to cause GI bleeding and related disturbances, including ulcers. Choline is an important counterion with minimal toxicity; it has been reported that choline salicylate demonstrated lower incidences of GI toxicity and was better tolerated at higher doses [43]. Therefore, the GI toxicity of APIs can be effectively reduced by the selection of appropriate salt forms.

### 4.2. Distribution and Clearance

Although not commonly utilized, salt formations have also been shown to affect the distribution and clearance of a drug molecule. Malek et al. demonstrated that the distribution properties of some antibiotics can be significantly altered by using macromolecular counterions [45]. Macromolecules such as polysaccharides, polyacrylic acids, sulfonic acids, and polyuronic acids were combined with popular antibiotics such as streptomycin and neomycin. Compared with streptomycin sulfate salt, these high molecular weight counterion salts with streptomycin showed a higher distribution of the drug to the lymph nodes, and less drug presence in the plasma. Selective distribution then resulted in the delayed clearance of streptomycin [46]. This opens up a great avenue for research to find unique macromolecular salt forms to alter the distribution of a drug based on the therapeutic outcomes desired.

### 4.3. Onset and Termination of Therapeutic Effects

Based on therapeutic indication, some drug formulations require a slower onset and termination of therapeutic effect. Different salt forms have been effectively utilized to alter the onset and duration of action of drugs. For example, it was observed that single salt amphetamine in dextroamphetamine preparations was not a good choice for fast and sustainable psychostimulant effects. Instead, Adderall XR^®^ was designed as a combination of the aspartate and sulfate salts of amphetamine, plus the saccharate and sulfate salts of dextroamphetamine. These different salts in a single drug product allowed different metabolism rates and possessed different onsets of action. This resulted in a faster induction of therapeutic effect while maintaining that effect for a sufficiently long time [47]. This example illustrates how salt can be utilized to modify the onset and duration of action of a drug to achieve desired results.

### 4.4. Counteracting Side Effects

Sometimes, counterions are used in such a manner that the side effects of the parent drug can be decreased by the counterion used. For example, the antihistamine salts of penicillin have been reported in the literature, since penicillin has the potential to elicit an allergic response in some patients. The main idea was to mitigate this allergic response of penicillin by using well-documented anti-allergic drugs [48]. Similarly, the intramuscular (IM) injection of benzylpenicillin was co-formulated with benzathine counterion. Benzathine is a well-known local anesthetic that numbs the IM injection site. This co-formulation decreases the pain associated with a very high IM depot dose of benzylpenicillin, which is required to treat certain conditions such as syphilis [49]. Another renowned example reflecting the use of counterions to minimize side effects is Dramamine^®^ (diphenhydramine + 8-chloro theophylline), where 8-chloro theophylline acts as a stimulant to counteract the drowsiness caused by diphenhydramine [50,51].

### 4.5. Drug Interactions

The presence of free acid/base forms or a particular counterion can have some clinically relevant drug interactions, particularly when it is co-manufactured or co-administered with other drugs. Prasugrel represents an important example of a drug interaction when co-administered with proton pump inhibitors (PPIs) [52]. Prasugrel is available as a hydrochloride salt, which was found to offer better absorption at higher gastric pH, when compared with the free base form. However, during the manufacturing of the drug, it has been found that the acid–base reaction can convert the salt form to the free base form, thus affecting pharmacokinetics. This is further complicated by the concurrent use of PPIs along with prasugrel, and co-administration can alter gastric pH as well as the salt to base ratio. So, bioequivalence studies with or without PPIs became clinically relevant. It was found that when prasugrel in different salt/base ratios was co-administered with lansoprazole, all forms exhibited a similar extent of absorption; however, the rate of absorption was found to be different [52]. This was a very important clinical outcome, since a high salt to base conversion significantly delayed the maximal platelet aggregation achieved by prasugrel, which is an important therapeutic goal following myocardial infarction. Thus, different salt forms as well as drug interactions can have important clinical implications [52].

## 5. Economic Considerations

### Intellectual Property (IP) Considerations

Over the years, various generic pharmaceutical manufacturers have tried to bring different salt forms of an approved API to gain entry into the market, even before the original patent had expired. On the contrary, innovative salt formulations have helped original patent holders to extend proprietary rights or give market exclusivity to a generic manufacturer. Some of the benefits offered by innovative salt forms that may deserve patent protection are simplified manufacturing procedures, more stable analogues, newer routes of administration, or a completely different therapeutic use [53].

One of the well-known examples is the request by Dr. Reddy’s Laboratories to gain market approval of amlodipine maleate, even before the patent expiration of amlodipine besylate. This plea was rejected in favor of the original patent. However, some manufacturers were successful by modifying certain dosage characteristics, as they demonstrated unique advantages. One well-known example is diclofenac epolamine (Flector^®^), which was approved and patented as a transdermal patch while its sodium and potassium salts were already available as generic tablets, capsules, topical gels, and solutions. The original patent on the traditional formulation was issued on 4 March 1997. The Institut Biochimique SA (IBSA) got a new patent on Flector^®^, which was approved by the FDA on 31 January 2007, and is valid until 13 April 2019 [54]. The innovative formulation justified the extended patent and marketing exclusivity. This demonstrates that patenting innovative salt forms can give market exclusivity to some products and help companies protect their intellectual properties. 

**Figure 4:**
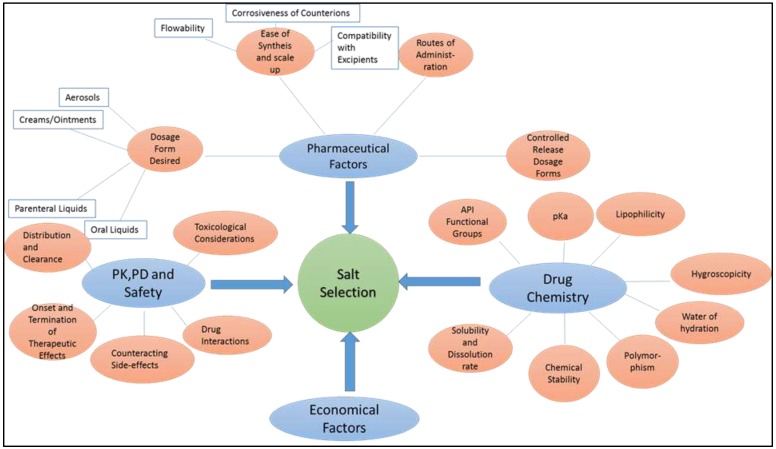
Various factors affecting the salt selection process.

All factors affecting the salt selection process are summarized in Figure 4 for clarity.

## 6. Screening, Preparation, and Characterization of Salts

There are various articles addressing the screening, preparation, and characterization of salts [55,56,57]. The intent of this section is not to provide an all-encompassing account of the processes involved, but rather to offer a brief summary for the purpose of tying loose ends among the selection, screening, preparation, and characterization of salts. The screening process of the salts starts with the selection of possible counterions to form the salt [9]. The salts of parent compounds (PC) are identified by screening an extensive number of salt forms (SFs) under a variety of crystallization conditions. Extensive screens are traditionally conducted at different conditions to identify salts with ideal properties for formulation development [55]. The drug must be completely ionized in a single state ionization in order to allow salt formation, since incomplete ionization can lead to the precipitation of the unionized form. As mentioned in the pK_a_ section, the ideal pK_a_ difference should be maintained in the solvent system that is used for crystallization; the challenge for this requirement is that often, non-aqueous or mixed solvent systems are used for crystallization, which can affect pK_a_. Recent advances such as a microfluidic platform can overcome the limitation of solvent incompatibility. The microfluidic platform is comprised of 48 wells, and each well is approximately 87.5 nL in volume. The reduction in volume enables salt screening for samples as low as 1 mg [55]. The salt of the API is prepared using one of four methods: thermal, anti-solvent, evaporation, and slurry conversion [9]. Regardless of the method employed, the crystallization is influenced by the additive type, concentration, pH, and ionic strength [9]. The prepared salt is characterized by X-ray powder diffraction, which gives information about whether the sample is crystalline or amorphous. Additionally, infrared (IR), Raman, and nuclear magnetic resonance (NMR) spectroscopy provides information about the interaction between the parent compound and the counterion [9]. Thermal methods such as differential scanning calorimetry (DSC) are used to determine the melting point and enthalpy of fusion. Both of those properties are helpful in predicting the solubility and stability of salts [9]. The purity and crystallinity of the sample can be assessed from the sharpness of the endotherm. The nature of the endotherm can also give information on the hydrates and solvates in the crystal structure [9]. Nievergelt et al. recently reported a high throughput screening of salts of cationic APIs using a semiautomatic technique. Their method used only nanoliters of the solution of the analyte for crystallization, yielding single crystals of appropriate size for characterization by a single-crystal X-ray structure determination [56].

## 7. Conclusions

The salt formation of an API is an integral part of the formulation development process. Traditionally, improving solubility is one of the fundamental reasons to employ salt forms. This article summarized that a unique salt form can have implications far from solubility. The choice of the ideal salt form can improve the solid-state properties of the API, and can ease the burden of time consumption and expensive formulation development. The counterions of the salts that are used can positively affect the applicability of the drugs in various dosage forms by improving the formulation properties. The appropriate salt form of the API is important in order to achieve the desired outcome, and can also have an immense economic impact.

## Figures and Tables

**Table 1:** List of currently available counterions for salt formation [9].

Chemistry (Type of Ion)	Examples of Counterions	
Cations	Aluminum Arginine Benzathine Calcium Chloroprocaine Choline Diethanolamine Ethanolamine Ethylenediamine	Lysine Magnesium Histidine Lithium Meglumine Potassium Procaine Sodium Triethylamine Zinc
Anions	Acetate Aspartate Benzenesulfonate Benzoate Besylate Bicarbonate Bitartrate Bromide Camsylate Carbonate Chloride Citrate Decanoate Edetate Esylate Fumarate Gluceptate Gluconate Glutamate Glycolate Hexanoate Hydroxynaphthoate Iodide Isethionate Lactate	Lactobionate Malate Maleate Mandelate Mesylate Methylsulfate Mucate Napsylate Nitrate Octanoate Oleate Pamoate Pantothenate Phosphate Polygalacturonate Propionate Salicylate Stearate Acetate Succinate Sulfate Tartrate Teoclate Tosylate

**Table 2:** Counterions of ibuprofen and their respective log P values and membrane absorption values.

Ibuprofen Counterion	Log P	Intestinal Flux (µg·cm^−1^·h^−1^)
Sodium	0.92	3.09
Ethylamine	0.97	5.42
Ethylenediamine	1.11	15.31
Diethylamine	1.12	7.91
Triethylamine	1.18	48.4

## Abbreviations

API	Active Pharmaceutical Ingredient
FDA	Food and Drug Administration
GRAS	Generally Regarded as Safe
IR	Immediate Release
PPI	Proton Pump Inhibitors
